# Supplementary material for: Comparing Inertial Measurement Units to Markerless Video Analysis for Movement Symmetry in Quarter Horses
Source: Sensors (Basel). 2023 Oct 12;23(20):8414. doi: 10.3390/s23208414 (PMC10610735; doi:10.3390/s23208414)
Supplement: Supplementary file 1 [file sensors-23-08414-s001.zip › sensors-2647706-supplementary.pdf]

sensors

Supplementary Materials

Additional illustration of the distribution of movement symmetry values across different groups of horses for the different exercise conditions. Horses were grouped either by patterns in their head movement symmetry (Figure S1) or their pelvic movement symmetry (Figure S2) during the starighť-line, hard surface assessment.

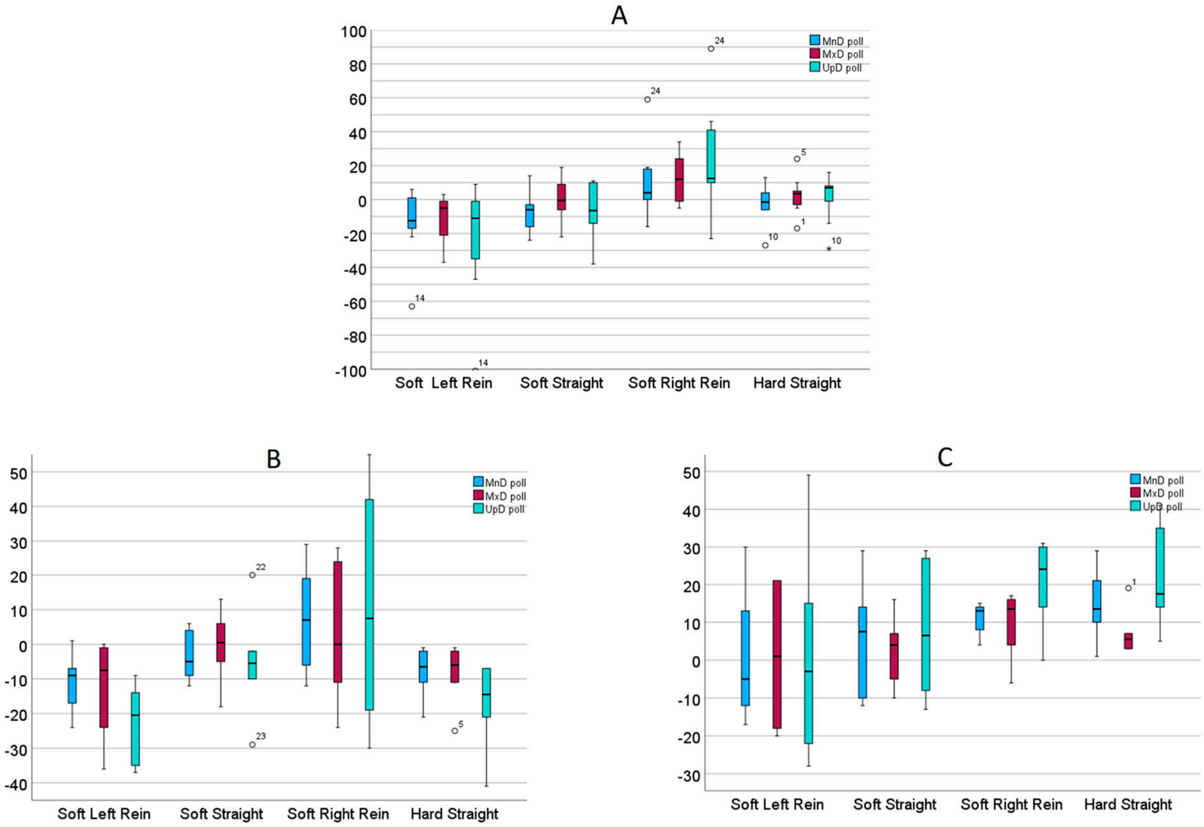

Figure S1. Box plots of movement asymmetry values for three groups of horses grouped according to **forelimb movement symmetry** patterns. Separate plots for combinations of movement direction (straight, left rein, right rein) and surface (hard, soft). Left and right rein circle exercise was only assessed on the soft surface. Negative values indicate 'left asymmetry', i.e. reduced force production with the **left forelimb** and positive values indicate 'right asymmetry', i.e. reduced force production with the right **forelimb**. Horses were grouped according to the movement symmetry measurements conducted on the hard surface during straight line trot. (**A**) 10 horses showing **mixed forelimb asymmetry** across the three head movement parameters; (**B**) 6 horses showing **left forelimb asymmetry** for all three head movement parameters; (**C**) 6 horses showing **right forelimb asymmetry** for all three head movement parameters.

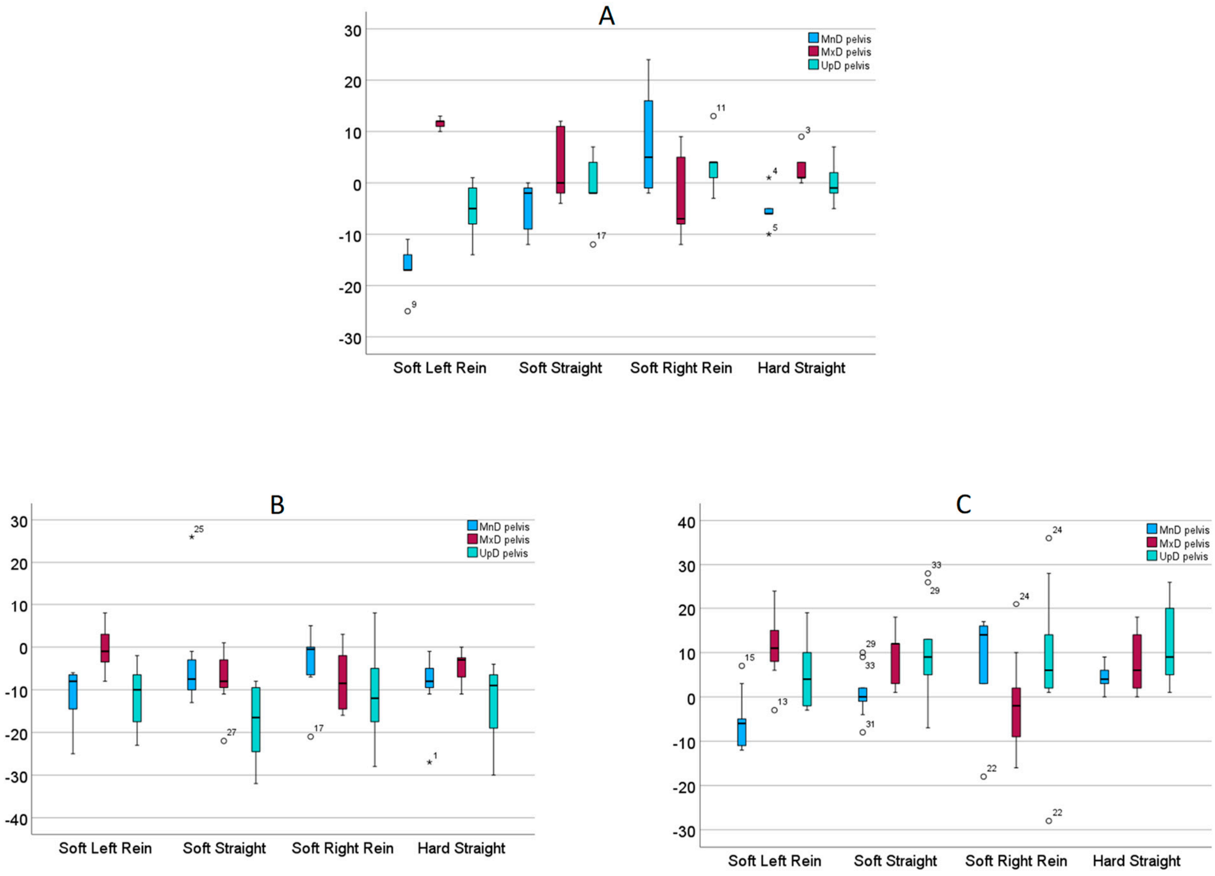

Figure S2. Box plots of movement asymmetry values for three groups of horses grouped according to hind limb movement symmetry patterns. Separate plots for combinations of movement direction (straight, left rein, right rein) and surface (hard, soft). Left and right rein circle exercise was only assessed on the soft surface. Negative values indicate 'left asymmetry', i.e. reduced force production with the left hind limb and positive values indicate 'right asymmetry', i.e. reduced force production with the right hind limb. Horses were grouped according to the movement symmetry measurements conducted on the hard surface during straight line trot. (**A**) 5 horses showing mixed hind limb asymmetry across the three pelvic movement parameters; (**B**) 8 horses showing left hind limb asymmetry for all three pelvic movement parameters; (**C**) 9 horses showing right hind limb asymmetry for all three pelvic movement parameters.

Quantile Quantile plots for illustration of effects related to proportional bias in Bland and Altman analysis:

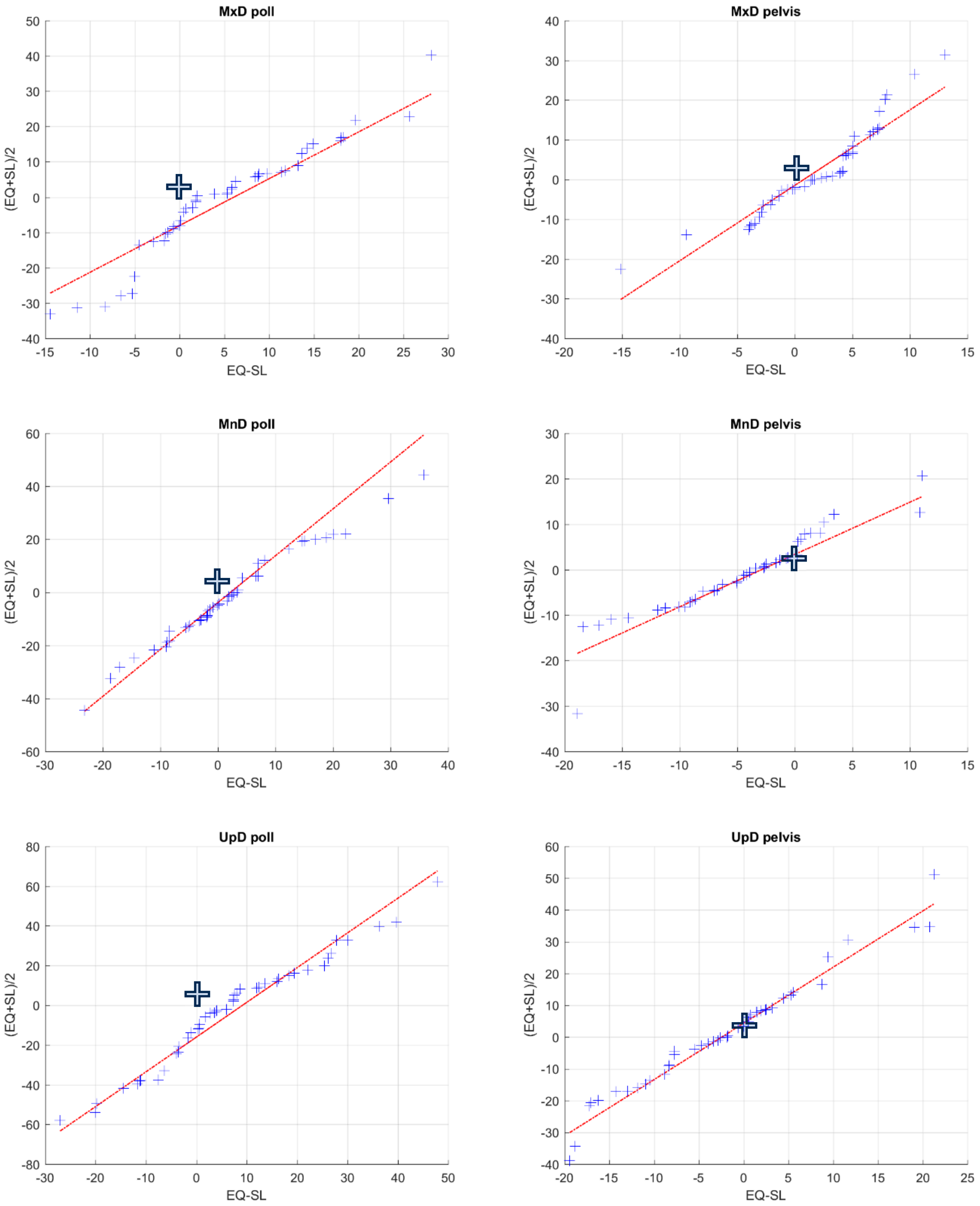

Figure S3. Quantile-quantile plots of between-system difference (x-axis, EQ-SL) against system average values (y-axis, (EQ+SL)/2) for three movement symmetry variables quantifying differences between vertical displacement minima (MnD), maxima (MxD) and upward movement amplitudes (UpD) between stride halves for straight-line trot from N=22 reining Quarter horses assessed on soft and hard ground. Movement symmetry variables are given for vertical head movement (poll) and vertical hind quarter movement (pelvis). EQ: EquiGait inertial sensor measurement; SL: Sleip video measurement.

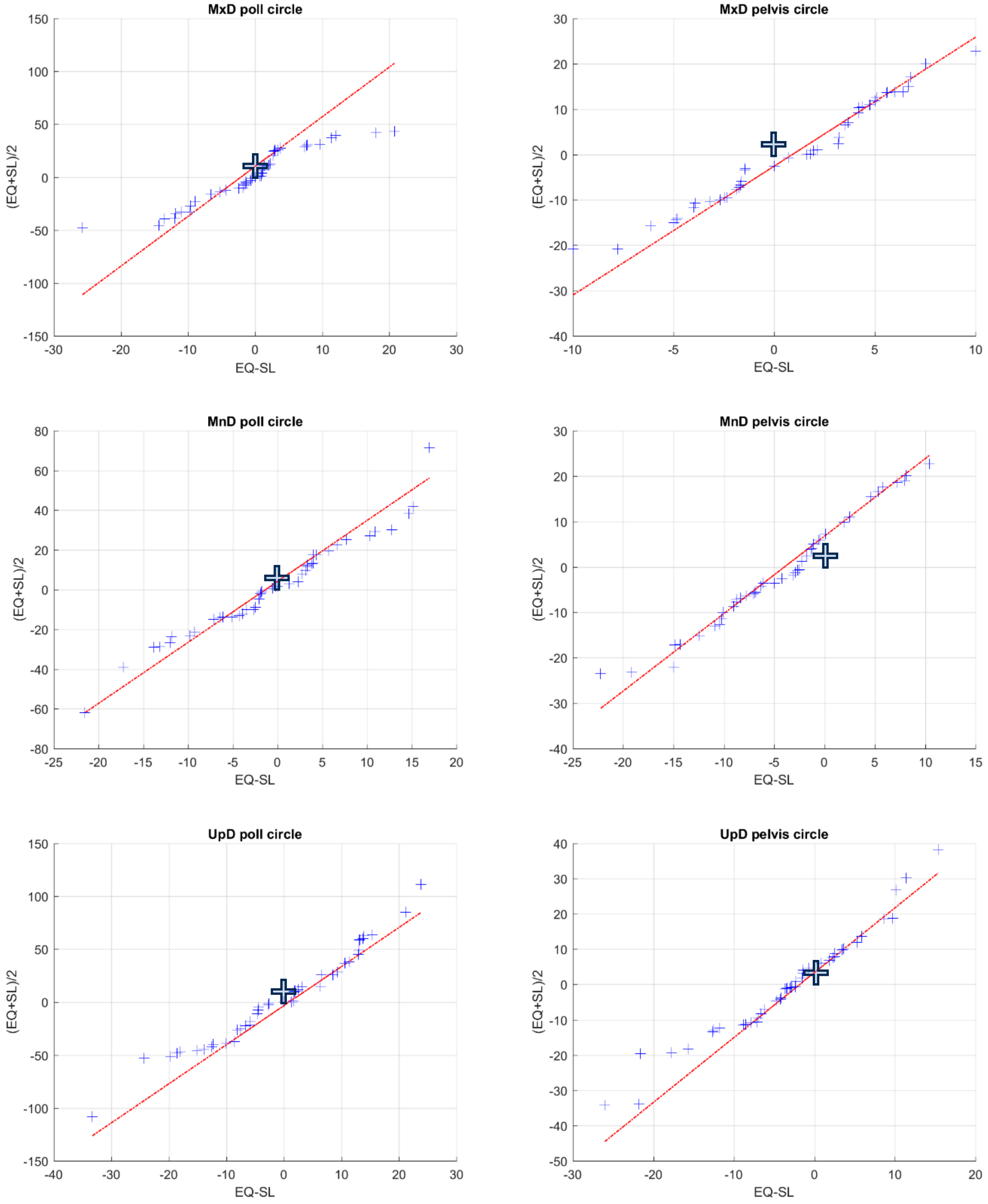

Figure S4. Quantile-quantile plots of between-system difference (x-axis, EQ-SL) against system average values (y-axis, (EQ+SL)/2) for three movement symmetry variables quantifying differences between vertical displacement minima (MnD), maxima (MxD) and upward movement amplitudes (UpD) between stride halves for circular trot (lunge) from N=22 reining Quarter horses assessed on soft and hard ground. Movement symmetry variables are given for vertical head movement (poll) and vertical hind quarter movement (pelvis). EQ: EquiGait inertial sensor measurement; SL: Sleip video measurement.
